# Institutional Housekeeping

**Published:** 1911-11-11

**Authors:** 


### INSTITUTIONAL HOUSEKEEPING.

(Contributions invited: Workers' Questions will be welcome.)

### Faults in Institution Menus.

There is a general consensus of opinion that institution meals leave much to be desired. Food of poor quality and badly-cooked food are both responsible for many complaints. But perhaps the crucial fault which the average meal partaken of by a mixed company of workers will show is a bad combination of otherwise excellent articles of diet. These unintelligent combinations are responsible for much impaired health, with a corresponding decrease of mental and physical vigour, not to mention actual disease. In planning the dietary it is necessary to take into account the habits of those composing the household, whether their brains are keenly exercised while their bodies are quiescent, whether mechanical muscular energy is principally needed, or whether both brain and body are kept continually on the alert. Moreover, it usually happens that in an institution the same dinner is shared by persons engaged in varied duties. Some will have been sitting still for four or five hours. Others will have been on their feet and are strained with fatigue, while some again may have a keen appetite after exercise in the open air. Yet all are required to assimilate food of the same type.

The ideal housekeeper, could we but find her, would know not only how to buy, plan, cook, and serve the food, but also the comparative food-value of each dish, and how best to distribute the various foodstuffs at her disposal. Not only would she regard the immediate effect on the appetites, digestions, and hence the tempers of those for whom she caters, but also what will be the future effect in building up the tissues of the body.

It is a well-known fact that various kinds of food are necessary to sustain life and health. But it is a popular English superstition from which dwellers in institutions suffer largely, that the only really important item of the daily bill of fare is a meat dish at every meal.

Proteids, fats, carbohydrates, mineral matters or salts, and water are the bases of all foods, and a good dietary must satisfy the requirements of the body without overloading those organs charged with converting food into the chemical constituents needed by the blood and tissues.

*Proteids* are found in meat, fish, eggs, cheese, beans, lentils, dried peas, etc.; and in lesser proportion in such starchy foods as whole-meal bread, macaroni, etc.

Even when meat is well chosen, well cooked and attractively served, too exclusive a meat diet draws heavily not merely on the purse but on the economy of the body, for the digestive system is unable to use an excess of nitrogen and a heavy strain is thrown on the kidneys and other organs in the elimination of what is not required. Little by little the housekeeper should demonstrate the attractiveness, nutritive value, and digestibility of the rival proteid foodstuffs to meat, taking due heed always that the meat-eater is not neglected. Savoury dishes prepared from cheese, macaroni, nuts, pulse, and certain cereals would prove a change and relief from the monotony of meat, and afford body-building materials in easily assimiliated form. The diners ought to have something to fall back upon of a nutritious character, in the not uncommon event of meat being presented to them which was originally of poor quality and has suffered injury at the hands of the cook.

*Fats* lie chiefly in the fat of meat and bacon, including dripping and lard; in butter and cream; and in salad oil. They are valuable articles of diet, for they consume readily in the body, and produce heat and energy. Fats also aid in the assimilation of other food. But burnt fat and foods fried in over-heated fat decompose into fatty acids, which give rise to that irritating form of digestion known as acidity or heartburn.

*Carbohydrates* are supplied by such food as potatoes, bread, rice, cornflour, jams, syrup, sugar, etc. It may be noticed here that although fats and carbohydrates both mainly supply heat and energy to the body, the same weight of the former class of food will yield two and a quarter times the amount of energy supplied by the latter. An average-sized lump of sugar has about the same food-value as an ounce of potatoes.

The institution dietary is particularly prone to err in its methods of combining the carbohydrates. Puddings of a luscious and stodgy nature figure largely in the ordinary menu and are usually popular, since they stay the appetite and satisfy a craving for sweet things. They are not, however, suitable for workers who must return to their duties without an interval for rest.

*Mineral matters* are principally taken unconsciously in their natural form as they occur in milk, eggs, water, fresh fruit, vegetables, etc., and should only be used very sparingly in the form of common salt added as a condiment. The absence of fresh fruit in institution dietaries is a serious blot.

*Water* is more essential to health than is often realised. It aids in carrying the nutritive materials through the body, and also cleanses it by aiding to rid it of its impurities and waste products. The natural craving for liquids is too frequently satisfied with tea, coffee, milk, or water flavoured (one should rather say adulterated) by acids or gases. It is seldom served in institutions in an appetising fashion, and a little trouble exerted to send it to table chilled and sparkling would make it what it ought to be, a really popular beverage. Where ice is plentiful the cost of cooling the water is negligible, and by the simple expedient of pouring it briskly from one jug to another several times the natural aeration can be restored when boiling is found necessary.

